# Diamond Open Access publishing practices among researchers in Ghana

**DOI:** 10.1371/journal.pone.0356546

**Published:** 2026-08-26

**Authors:** Dominic Dankwah Agyei, Philip Larry Anguah, Christopher Ayisah, George Clifford Yamson, Fred K. Hayibor, Patrick Ngulube

**Affiliations:** 1 University Library, University of Health and Allied Sciences, Ho, Volta Region, Ghana; 2 University Library, Ghana Communication Technology University, Accra, Greater Accra Region, Ghana; 3 Fred N. Binka School of Public Health, University of Health and Allied Sciences, Ho, Volta Region, Ghana; 4 University Library, University of Environment and Sustainable Development, Somanya, Eastern Region, Ghana; 5 University Library, University of Health and Allied Sciences, Ho, Volta Region, Ghana; 6 School of Interdisciplinary Research & Graduate Studies (SIRGS), University of South Africa, Pretoria, South Africa; Thammasat University, THAILAND

## Abstract

The rise of Diamond Open Access (DOA) represents a major transformation in scholarly communication, redefining traditional systems of knowledge distribution. Diamond open access model has gained recognition as the most equitable and sustainable form of open access publishing. This study aimed to assess the awareness, perception and knowledge of DOA publishing practices among researchers in Ghana. A cross-sectional study design was used to conveniently collect data from 237 researchers across various institutions in Ghana. Data were collected using structured questionnaires Via Microsoft Forms and analysed using STATA version 17. Descriptive statistics, t-tests, ANOVA, factor analysis, Fisher’s exact test and logistic regression were conducted to identify patterns and relationships in the data. Statistical significance was set at p < 0.05 with a 95% confidence interval. Over half of the participants had no prior experience with Open Access (OA) publishing whilst about two-third of them had never heard of DOA. Only a small proportion could correctly identify the CC BY license, reflecting limited understanding of open licensing. Most of the respondents demonstrated poor knowledge of DOA, with many knowing about the concept through peers and informal networks. Knowledge of DOA was significantly associated with age, type of institution, current rank, and level of academic qualification. Perceived benefits of DOA, particularly regarding visibility and dissemination, differed significantly by current rank and academic qualification. The findings underscore the need for strong policy and institutional reforms to enhance awareness and adoption of DOA in Ghana. Universities and research institutions should develop clear OA and DOA policies, integrate open-access education into research training, and establish sustainable funding mechanisms to support equitable publishing. Strengthening library led advocacy, creating institutional repositories, and fostering national collaboration among academic bodies and policymakers are vital to promote transparency, inclusivity, and global visibility in scholarly communication.

## Introduction

In recent times, academia has experienced a significant transformation influenced by the Open Access Publishing (OAP) model, which provides diverse avenues for disseminating research findings globally [1]. Open access publication emerged in the 21st century as a collaborative initiative between scholars and publishers. Before its introduction, university researchers seeking tenure, promotion, or professional recognition often submitted their scholarly works to commercial journals without compensation [2]. Although scholars continued to provide free manuscripts to enhance their academic standing, journal subscription fees have sharply increased over the years [3]. According to Moore (2025), the concept of open access arose in response to scholars’ protests in North America and Britain against the increasing cost of journals and the exploitative nature of traditional subscription-based publishing systems [4].

Despite efforts to enhance accessibility, a large portion of scholarly literature remains behind expensive paywalls. As a result, open access publishing was built on the belief that the outcomes of academic research should benefit society and that scholarly works should be freely accessible to all. Open access publishers employ different approaches to make academic content available at little or no cost to users. While OAP offers numerous benefits, it also faces significant challenges [5]. To overcome these challenges, three main models; Green, Gold, and Diamond (also known as Platinum) have emerged to promote equitable access to research outputs. However, Green open access (self-archiving) involves making published or pre- and post-print versions of research freely available in repositories or on personal websites. Gold open access refers to publishing in open access journals or platforms where articles are immediately accessible and undergo standard peer review, whereas Diamond open access is a subtype of gold open access that does not require publication fees, as it is typically supported by non-commercial, community-driven funding models [6–8]. Each model seeks to make knowledge freely available but operates differently. The Gold and Green models, however, still involve some form of payment, with the Gold through article processing fees and Green through costs associated with immediate or peer-reviewed access [5].

Among these, the diamond open access (DOA) model has gained recognition as the most equitable and sustainable form of open access publishing [9]. This model eliminates fees for both authors and readers, relying instead on financial support from universities, research institutions, or philanthropic organisations [9,10]. The rise of DOA represents a major transformation in scholarly communication, redefining traditional systems of knowledge distribution [10]. It provides unrestricted and immediate access to digital research outputs, ensuring that scholarly work is freely available to everyone, regardless of socioeconomic status. By removing financial barriers, diamond open access expands the reach of research, enhances authors’ visibility, increases citation potential, and strengthens the overall impact of academic work for the public good [9].

In the Ghanaian context, there is a pressing need to understand researchers’ knowledge and perception regarding the use of diamond open access for scholarly dissemination, given the paucity of local studies. Limited discussions exist on the benefits and challenges influencing researchers’ willingness to publish in DOA journals. For instance, previous studies indicate that although most Ghanaian researchers were aware of open access and used OA resources in their work, only a small proportion published in OA journals, even though all participants recognised and benefited from its role in disseminating scientific information [11,12]. Their hesitation stemmed from concerns about peer-review quality, copyright integrity, and ethical safeguards in open access publishing [11]. Furthermore, a comprehensive search of literature from Google Scholar and Scopus on DOA in Ghana supported the notion of the lack of evidence.

This study, therefore, provides valuable insights into Ghanaian researchers’ awareness, perceptions, and knowledge of DOA publishing. It explores the perceived benefits, challenges, and factors influencing the adoption of DOA for disseminating scientific knowledge. By addressing this gap, the study aims to inform institutional policies and promote best practices that encourage equitable access to research through DOA.

### Literature review

Open Access (OA) represents a significant change in scholarly communication, prioritising the unrestricted and free availability of research outputs online, free from financial or legal barriers [13]. This paradigm shift emerged as a response to the limitations of traditional subscription-based publishing models, which often restrict knowledge dissemination and perpetuate global information inequities [14]. The OA movement seeks to democratise knowledge access, enhance research visibility, and amplify the societal and academic impact of scholarship through more transparent and inclusive publishing systems [12]. The intellectual basis of OA is centred on enhancing transparency, validity, replicability, and accessibility in scientific communication [15]. Traditional publishing models have been criticised for fostering financial exclusion, as authors produce work without remuneration, while readers and institutions face costly subscription fees and large “big deal” contracts to access publications [16,17]. These systemic barriers have intensified calls for more equitable and sustainable models of knowledge sharing. In response, global and national initiatives since the early 2000s have established policy frameworks supporting OA dissemination across research institutions, funding agencies, and governments [18].

However, despite its promise, OA publishing faces notable challenges. Article Processing Charges imposed by many gold OA journals hinder participation, particularly among researchers in low- and middle-income countries and within the social sciences and humanities [19]. Institutions encounter challenges concerning repository maintenance, policy enforcement, and copyright issues. The rise of predatory journals, which emphasise profit over stringent peer review, has generated scepticism and concerns among researchers regarding open access pathways [20]. Africa’s contribution to global scientific output remains disproportionately low, providing only about 1.3% of worldwide publications with most research concentrated in South Africa, Nigeria, Kenya, and Egypt [21,22]. Research literature identifies this gap as a result of systemic constraints, insufficient funding, and publication obstacles within African research ecosystems [23,24]. Financial barriers, especially pyament of APCs, remain a significant impediment, limiting the involvement of African researchers in global open access publishing and sustaining publication disparities between the Global North and South.

In light of these challenges, DOA has emerged as a promising alternative. Unlike gold OA, DOA eliminates both reader and author fees, thereby addressing the inequities associated with APC based publishing [25,26]. This model, often community-driven and institutionally supported, emphasises non-commercial academic collaboration and equitable access to scholarly content. Established platforms such as Érudit, OpenEdition, and SciELO exemplify sustainable DOA initiatives worldwide [27]. Diamond Open Access empowers researchers to play an active role in the publication process, ensuring that their work remains accessible and socially impactful [28]. With publication costs continuing to rise in commercial gold OA journals, DOA offers a sustainable and inclusive alternative [29]. Studies estimate that between 17,000 and 29,000 DOA journals exist globally, publishing approximately 356,000 articles annually, representing nearly 9% of global scholarly output [30]. Regional disparities persist, with Latin America leading globally in DOA adoption [31].

Nonetheless, awareness and knowledge of diamond open access models are still restricted in numerous low- and middle-income research environments. In Africa, awareness and understanding of the DOA model remain limited. While 78% of academic librarians have published in open access journals and 73.45% view OA journals positively [32], about 74.6% remain unfamiliar with DOA publishing [33]. Furthermore, more than half report financial constraints that limit their ability to publish even in open-access outlets [32]. Similarly, researchers in Angola and other African countries exhibit minimal understanding of the DOA model [34].

The limited awareness and adoption of DOA in Africa underscore a critical need for advocacy, capacity-building, and policy interventions to strengthen researchers’ engagement with equitable publishing models. DOA holds immense potential for promoting regional scholarship, bridging global research disparities, and advancing the visibility of African knowledge production. To date, there is a scarcity of empirical studies examining the awareness, perception, and knowledge of diamond open access publishing practices among researchers in Africa and to the best of current knowledge, this study is the first of its kind conducted in Ghana.

## Methods

### Study design and setting

A cross-sectional study design was used to estimate population parameters such as researchers’ awareness, perceptions, and knowledge of DOA publishing practices and the average value of a quantitative variable among researchers. The research design allows for the collection and statistical analysis of data to identify patterns and correlations in the participants’ responses [35]. The research was conducted among Ghana researchers in various institutions across the country.

### Sampling procedure and techniques

This study used the convenience sampling technique to collect data from 237 researchers from Ghanaian researchers on their knowledge and experiences of DOA publishing practices. Convenience sampling was chosen due to geographical proximity, availability at a given time, or willingness to participate in the research.

### Data collection tool and procedure

The study adopted a quantitative survey design. A structured questionnaire was created based on insights from literature reviews and expert opinions. The initial version of the instrument was pre-tested among 10 faculty members from a medical university in Ghana to evaluate its clarity, wording, and structure. Feedback from the pre-test was used to refine the questionnaire, ensuring that all items were clear, relevant, and easy to understand. Reliability was acceptable with Cronbach’s alpha of 0.87. The final version was created using Microsoft Forms, and the survey link was shared across academic platforms to reach researchers from different institutions. Data collection occurred from May to August 2025.

### Ethical considerations

Ethical approval for the study was obtained from the Research Review Committee of the University of Health and Allied Sciences (UHAS-REC), with clearance number UR107/0326. Prior to participation, informed consent was obtained electronically through the Microsoft Forms online survey platform after participants had read the study information and agreed to take part. Confidentiality was strictly maintained, as no personally identifiable information such as names was collected. Instead, unique codes were assigned to responses, and access to the dataset was restricted to the research team only.

### Study variables and measure

The main dependent variables included knowledge of diamond open access journals, as well as perceived benefits and challenges of DOA publishing practices. Knowledge about DOA journals was measured with two variables: “Heard the term Diamond Open Access” and “Characteristics of Diamond Open Access Journals.” Correct responses were summed and categorised based on a median split of their composite scores, categorising knowledge as good (≥50% of total) or poor (<50%). For perceived benefits and challenges, factor analysis was used to identify underlying latent constructs within the items, grouping related variables into coherent factors. This approach reduced data complexity and improved the interpretability of the constructs. The independent variables comprised participants’ background characteristics, including age, institution type, gender, academic rank, qualification, and years of experience.

### Data analysis

The collected data were exported to Stata version 17 for cleaning and analysis. Data cleaning involved the removal of duplicates and errors to ensure accuracy. Descriptive statistics were used to summarise sociodemographic characteristics of the respondents. A t-test was conducted to compare the perceived benefits of DOA across respondents’ demographic characteristics. Furthermore, ANOVA was used to examine differences in perceived benefits and challenges of DOA publishing among respondents. To examine the perceived benefits and challenges of DOA publishing, an exploratory factor analysis was conducted to determine the underlying structure of items measuring these constructs. Factors were extracted using principal component analysis, and a rotation method was applied to improve interpretability. The number of factors retained was determined by eigenvalues greater than 1 and by inspection of the scree plot. Items with factor loadings of at least 0.40 were retained within their respective factors. Associations between categorical variables were assessed using Fisher’s exact test. Further, bivariate logistic regression was conducted to examine crude associations between knowledge of DOA publishing practices and demographic characteristics. All variables in the bivariate analysis were included in the multivariable logistic regression to estimate adjusted odds ratios (AORs). The Hosmer-Lemeshow test yielded a p-value of 0.4330, indicating a good fit of the logistic regression model, while Variance Inflation Factor (VIF) assessments showed no evidence of multicollinearity. Statistical significance was determined at p < 0.05, and a 95% confidence interval was used to estimate the precision of the results.

## Results

### Sociodemographic characteristics of respondents

Table 1 details out the sociodemographic characteristics of the respondents. The study showed that more than half of the respondents 124 (52.3%) were aged 18–34 years with a mean age of 33.9 ± 11.5. Most of the respondents worked in universities 188 (79.3%) and most of them were males 142 (59.9%). Nearly half 108 (45.6%) were lecturers or assistant librarians, while (38.8%) were graduate students. Regarding academic qualification most of them either had master’s degree 86 (36.3%) or a PhD 55 (23.2%) PhDs.

**Table 1:** Sociodemographic characteristics of participants.

Variable	Frequency (n = 237)	Percentage (%)
**Age mean (±SD)**	33.9 (±11.5)	
18-34	124	52.3
35-49	92	38.8
50-60	19	8.0
+61	2	0.9
**Type of Institution**		
Universities	188	79.4
Colleges	22	9.3
Medical centres	7	2.9
Others	20	8.4
**Gender**		
Female	95	40.1
Male	142	59.9
**Current Rank**		
Professor	11	4.7
Lecturer/Assistant Librarian/Research Fellows	108	45.6
Graduate student	92	38.8
Others	26	10.9
**Level Of Academic Qualification**		
Bachelor	88	37.1
Master	86	36.3
PhD	55	23.2
Post doctoral	8	3.4
**Years Of Experience**		
None	8	3.4
1-10yrs	201	84.8
11-20yrs	26	10.9
21-30yrs	2	0.9

### Awareness and understanding of Diamond Open Access journals

One of the main aims of this study was to explore participants’ awareness and experience with OA and DOA. To this end, it was found out that about one-third 79 (33.3%) had never published, with slightly more than half 122 (51.5%) having no prior experience with OA publishing. Further, it was realised that over half 124 (52.3%) of the respondents reported having no publications under OA, whereas 63 (26.6%) had between 1–4 OA publications. Also, it was noted that only 84 (35.4%) of the respondents were aware of DOA publishing practices. Regarding knowledge of creative commons licenses, it was noted that only 31 (13.1%) correctly identified CC BY as the most common cc attribution, whilst 148 (62.5%) reported no knowledge. Table 2 gives further details.

**Table 2:** Awareness and understanding of Diamond Open Access journals.

Variable	Frequency (n = 237)	Percentage (%)
**Number of publications**		
No publications	79	33.3
1-4	88	37.1
5-9	28	11.8
10-19	18	7.6
20+	24	10.1
Experience with open access publishing	115	48.5
**Number of OA publications**		
None	124	52.3
1-4	63	26.6
5-9	24	10.1
10-19	17	7.2
20+	9	3.8
**Heard of the term Diamond Open Access**		
No	153	64.6
Yes	84	35.4
**DOA commonly preferred creative commons license**		
CC BY	31	13.1
CC BY-NC	12	5.1
CC BY-NC-ND	13	5.5
CC BY-NC-SA	7	2.9
CC BY-ND	7	2.9
CC BY-SA	11	4.6
CC0	8	3.4
Not Applicable/I do not know	148	62.5

### Key characteristics and source of information of Diamond Open Access journals

Regarding the features of DOA practices, it was noted that over half (55.9%) of the respondents identified free online accessibility as the main feature, followed by no author-processing charges (32.4%) and peer-reviewed publications (29.4%). About 26.5% noted immediate and unrestricted access to articles, whilst almost half of them 47.1% were unaware of DOA characteristics. Concerning those who indicated knowledge of DOA, it was noted peers and informal networks (14%) and academic or research sources (14%) were the main sources of information as in S1 and S2 Figs.

### Knowledge of Diamond Open Access publishing practices

**Fig 1:**
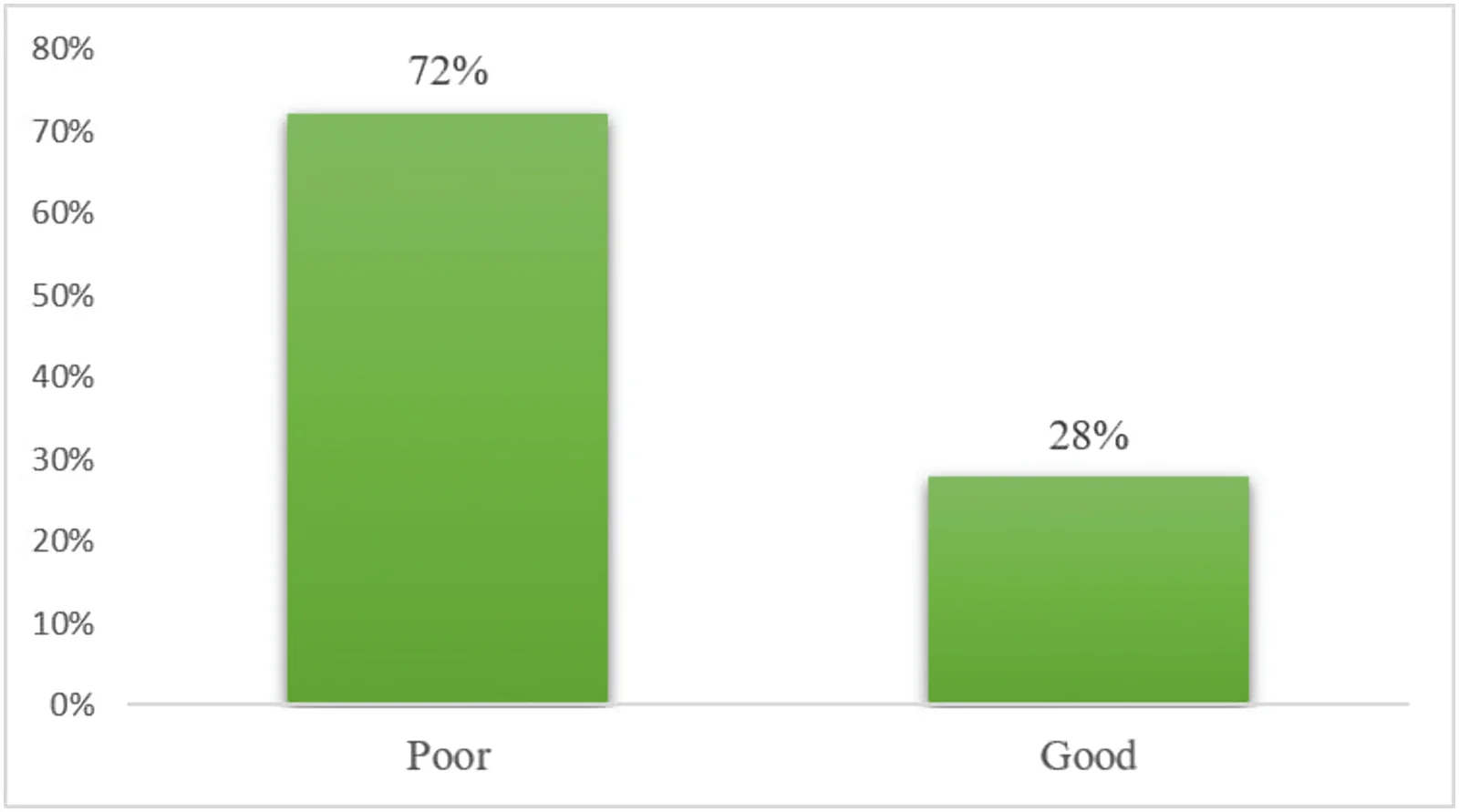
Knowledge of Diamond Open Access publishing practices.

Fig 1 revealed that, majority of participants 171 (72.2%) had poor knowledge of diamond open access publishing practices while 66 (28%) had good knowledge.

### Association between knowledge of Diamond Open Access and sociodemographic factors

This study made efforts to establish relationships between the sociodemographic characteristics of respondents and their knowledge of DOA publishing practices. To this end, it was realised that age (p = 0.003), type of institution (p = 0.027), current rank (p = 0.028), and level of academic qualification (p < 0.001) showed significant associations with knowledge as shown in Table 3.

**Table 3:** Association between knowledge of Diamond Open Access and socio-demographic factors.

Variable	Knowledge of Diamond Open Access		Fisher’s Exact Test Stat.
	Poor 171 (72.2%)	Good 66 (27.9%)	
**Age**			
18-34	98 (79.0)	26 (20.9)	0.003
35-49	57 (61.9)	35 (38.0)	
50-60	16 (84.2)	3 (15.8)	
61+	0 (0.0)	2 (100.0)	
**Type of institution**			
Universities	128 (68.1)	60 (31.9)	0.027
Colleges	18 (81.8)	4 (18.2)	
Medical centres	6 (85.7)	1 (14.3)	
Others	19 (95.0)	1 (5.0)	
Gender			
female	73 (76.8)	22 (23.2)	0.121
male	98 (69.0)	44 (30.9)	
**Current rank**			
Professor	7 (63.6)	4 (36.4)	0.021
Lecturer/Assistant Librarian/Research Fellows	69 (63.9)	39 (36.1)	
Graduate student	76 (82.6)	16 (17.4)	
Others	19 (73.1)	7 (26.9)	
**Level of academic qualification**			
Bachelor	70 (79.6)	18 (20.5)	<0.001
Master	70 (81.4)	16 (18.6)	
PhD	25 (45.5)	30 (54.6)	
Post doctoral	6 (75.0)	2 (25.0)	
**Years of experience**			
None	5 (62.5)	3 (37.5)	0.185
1-10yrs	150 (74.6)	51 (25.4)	
11-20yrs	15 (57.7)	11 (42.3)	
21-30yrs	1 (50.0)	1 (50.0)	

### Factors influencing knowledge of DOA and sociodemographic factors

In an effort to explore the factors associated with knowledge of DOA publishing practices, crude and adjusted odds ratios (cOR and aOR) were conducted. After adjusting for potential confounders, level of academic qualification remained significantly associated with knowledge. Participants with PhDs were 3.5 times more likely to have good knowledge compared to those with a bachelor’s degree (aOR = 3.52, 95% CI: 1.11–11.18, p = 0.033). Again, it was realised that age (χ², cOR: 2.31, 95% CI: 1.27–4.23, p = 0.006), type of institution (cOR: 8.91, 95% CI: 1.16–68.1, p = 0.035), and current rank showed significant associations in the crude model. However, these associations were not significant after adjustment. Table 4 gives further details.

**Table 4:** Factors influencing knowledge of Diamond Open Access and socio-demographic characteristics.

Variable	Knowledge of DOA publishing practices		cOR (95% CI), p-value	aOR (95% CI), p-value
	Poor 171 (72.2%)	Good 66 (27.9%)		
**Age**				
18-34	98 (79.0)	26 (20.9	1	1
35-49	57 (61.9)	35 (38.0)	2.31 (1.27-4.23), 0.006	0.92 (0.34-2.48), 0.878
50-60	16 (84.2)	3 (15.8)	0.71 (0.19-2.61), 0.603	0.23 (0.45-1.19), 0.080
61+	0 (0.0)	2 (100.0)	__	__
**Type of Institution**				
Others	19 (95.0)	1 (5.0)	1	1
Universities	128 (68.1)	60 (31.9)	8.91 (1.16-68.1), 0.035	7.48 (0.92-60.62), 0.60
Colleges	18 (81.8)	4 (18.2)	4.22 (0.43-41.45), 0.216	4.46 (0.40-49.52), 0.223
Medical centres	6 (85.7)	1 (14.3)	3.17 (0.17-58.70), 0.439	3.93 (0.19-78.77), 0.371
**Gender**				
Female	73 (76.8)	22 (23.2)	1	1
Male	98 (69.0)	44 (30.9)	1.49 (0.82-2.70), 0.189	1.14 (0.57-2.25), 0.714
**Current rank**				
Professor	7 (63.6)	4 (36.4)	1	1
Lecturer/Assistant Librarian/Research Fellows	69 (63.9)	39 (36.1)	0.99 (0.27-3.59), 0.987	0.68 (0.16-2.91), 0.608
Graduate student	76 (82.6)	16 (17.4)	0.37 (0.09-1.41), 0.145	0.44 (0.09-2.23), 0.319
Others	19 (73.1)	7 (26.9)	0.64 (0.14-2.89), 0.567	0.77 (0.12-5.09), 0.785
**Level of academic qualification**				
Bachelor	70 (79.6)	18 (20.5)	1	1
Master	70 (81.4)	16 (18.6)	0.89 (0.42-1.88), 0.758	0.96 (0.38-2.41), 0.926
PhD	25 (45.5)	30 (54.6)	4.67 (2.22-9.79), 0.000	3.52 (1.11-11.18), 0.033
Post doctoral	6 (75.0)	2 (25.0)	1.29 (0.24-6.97), 0.762	0.83 (0.13-5.34), 0.847
**Years of experience**				
None	5 (62.5)	3 (37.5)	1	1
1-10yrs	150 (74.6)	51 (25.4)	0.57 (0.13-2.46), 0.448	0.56 (0.09-3.57), 0.539
11-20yrs	15 (57.7)	11 (42.3)	1.22 (0.24-6.23), 0.809	0.66 (0.08-5.40), 0.702
21-30yrs	1 (50.0)	1 (50.0)	1.67 (0.07-37.73), 0.748	__

### Factor analysis of perceived benefits

In order to establish factors related to perceived benefits of DOA publishing practices, the Kaiser-Meyer-Olkin (KMO) and Bartlett’s test were conducted. A KMO value of 0.899 was obtained, indicating excellent sampling adequacy for factor analysis. Bartlett’s test of sphericity yielded a chi-square value of 1003.183 with 45 degrees of freedom and a significance level of 0.000, confirming that the variables are sufficiently intercorrelated and suitable for factor analysis. S3 Table presents the factor analysis results for “Visibility and Dissemination”. The factor recorded a strong reliability (Cronbach’s α = 0.8819) and explained 5% of the total variance (eigenvalue = 5.00179). Key items with high loadings included promoting global equity in scholarly communication (0.819), increasing citation potential (0.778), and fostering research innovation (0.744), highlighting DOA’s role in enhancing visibility and knowledge sharing.

### Factor analysis of perceived challenges

A similar test conducted for factors relating to perceived challenges of DOA publishing practices resulted in a KMO value of 0.908, indicating excellent sampling adequacy for factor analysis. Bartlett’s test of sphericity produced a chi-square value of 679.931 with 45 degrees of freedom and a significance level of 0.000, a confirmation that the variables are sufficiently intercorrelated for factor analysis. The determinant value of 0.053 further supports the suitability of the data. S4 Table shows the factor analysis results for “Quality Concern.” The factor accounted for 43.12% of the variance (eigenvalue = 4.31155) with high reliability (Cronbach’s α = 0.8819). Major factor loadings included lack of awareness of DOA (0.7418), technological and infrastructural challenges (0.7271), and sustainability issues (0.6371).

### ANOVA analysis of perceived benefits and challenges of Diamond Open Access publishing based on sociodemographic variables

Attempts were made to compares male and female participants’ mean scores on the perceived benefits and challenges of DOA publishing practices. For visibility and dissemination, males reported slightly lower perceived benefits (M = 2.2842) than females (M = 2.3021), with no significant difference (t = −0.186, p = 0.8523). For Quality concerns, males (M = 2.6126) scored marginally higher than females (M = 2.4831), though this difference was also not statistically significant (t = 1.392, p = 0.1653). S5 Fig gives further details.

### ANOVA analysis of perceived benefits of Diamond Open Access publishing based on sociodemographic variables

An ANOVA test on the perceived benefits of DOA publishing practices across demographic characteristics was conducted. Regarding visibility and dissemination, significant variations were observed in current rank (F = 5.01, p = 0.002) and academic qualification (F = 3.08, p = 0.028). Professors (mean = 2.845) and post-doctoral holders (mean = 2.538) exhibited higher perceived benefits, suggesting that individuals with higher academic standing tend to view DOA publishing practices more positively as shown in S6 Table.

### ANOVA analysis of perceived challenges of DOA publishing based on sociodemographic variables

Table 5 presents the ANOVA results on perceived challenges of DOA publishing across sociodemographic variables. For quality concerns, significant differences were found in current rank (F = 2.74, p = 0.0438), where professors (mean = 3.00) reported the highest challenges. Although age differences approached significance (F = 2.62, p = 0.0517), participants aged 60 and above (mean = 3.25) perceived greater challenges.

**Table 5:** ANOVA analysis of perceived challenges of DOA publishing based on demographic variables.

Quality concerns			
Variable	Mean	F-value	p-Value
**Age (years)**			
18–34	2.49	2.62	0.0517
35–49	2.51		
50–60	2.90		
Above 60	3.25		
**Type of institution**			
University	2.55	0.35	0.7909
Colleges	2.41		
Medical Centres	2.41		
Others	2.54		
**Gender**			
Male	2.61	1.94	0.1653
Female	2.48		
**Current Rank**			
Graduate Student	2.43	2.74	0.0438
Lecturer/Assistant Lecturer	2.54		
Professor	3.00		
Others	2.69		
**Highest Academic Qualification**			
Bachelor’s degree	2.48	1.09	0.3550
Master’s degree	2.58		
PhD	2.60		
Post-doctoral	2.19		

## Discussion

This study assessed researchers’ awareness, perceptions, and knowledge of DOA publishing practices in Ghana. It further explored the perceived benefits, challenges, and factors influencing the adoption of DOA for disseminating scientific knowledge. The findings demonstrate that whilst participants showed a general understanding of OA concepts, awareness and knowledge of the DOA publishing route remain limited. This aligns with earlier studies conducted across Africa, indicating that academic communities are more familiar with green and gold OA pathways than with diamond models [1,33]. A similar study conducted in Ghana reported low levels of OA knowledge among Ghanaian researchers, suggesting persistent knowledge gaps within local academic environments [11]. This implies that limited DOA knowledge may exacerbate existing publication inequalities, particularly in settings where APCs are a significant barrier to the dissemination of research output. Again, lack of institutional support, infrastructural deficiencies, and uncoordinated policies in local academic institutions could account for the low knowledge and adoption of DOA publishing practices.

Although participants acknowledged key characteristics of DOA such as free online accessibility and the absence of APC charges, many failed to accurately define the model and differentiate it from other OA pathways. This finding is similar to global findings showing uneven understanding of DOA despite increasing discussions about equitable scholarly communication [28]. Nevertheless, a number of participants recognised the benefit of DOA, including wider visibility and removal of APCs, which makes it consistent with existing studies revealing that DOA enhances research accessibility and supports equitable participation in global knowledge production [10,25].

A key finding of this study is the significant knowledge variation by academic qualification, with PhD holders demonstrating clearer understanding of DOA concepts than bachelor-level academics. Although prior OA research has highlighted demographic and disciplinary variations [36–38], the distinct influence of academic qualification on DOA awareness represents a novel contribution, especially within the African scholarly context. This suggests that early career researchers and junior faculty remain disadvantaged in accessing and utilising equitable publishing models.

Contrary to studies emphasising the central role of academic libraries in OA support and advocacy [14], participants in this study primarily relied on peer and informal networks for DOA information. This implies that limited institutional advocacy frameworks, reflecting earlier observations of weak OA and DOA policy uptake and awareness infrastructure in African settings [21,28]. The finding underscores the need for stronger institutional leadership and library-driven capacity-building to bridge knowledge and practice gaps. It is imperative for universities and research institutions to strengthen DOA education and policy structures to ensure researchers benefit from equitable publishing options. Moreover, early-career researchers require targeted support and training to enhance awareness, confidence, and utilisation of DOA pathways.

The study’s unique contribution lies in its empirical analysis of perceived benefits and challenges associated with DOA among Ghanaian researchers. Although prior scholarship has highlighted the potential of DOA to reduce publishing inequities and strengthen African research visibility [22,23], limited empirical work has explored how this perceived benefit and challenges vary across researcher demographics. This study fills that gap by systematically analysing demographic differences in perceived benefit and challenges.

The study revealed significant differences by academic rank and qualification, with professors and post-doctoral scholars perceiving greater benefits, suggesting that senior academics appreciate DOA’s potential for visibility and dissemination. However, the same groups expressed the highest quality concerns, alongside older participants (≥60 years), reflecting scepticism toward DOA models. This dual perception indicates that while senior scholars acknowledge DOA’s advantages, they remain cautious about its operational robustness and academic legitimacy. Such generational divides may stem from entrenched prestige driven publishing norms and limited sensitisation efforts targeting early career scholars [39]. These patterns imply that resistance to DOA may not stem solely from structural limitations, but also from entrenched academic cultures, prestige-oriented publishing norms, and limited targeted sensitisation efforts for early career researchers. Again, the rise of predatory journals, which emphasise profit over stringent peer review, is noted to have increased scepticism and concerns among researchers regarding DOA publishing practices [20].

These fears align with existing critiques suggesting operational vulnerabilities and visibility challenges within DOA publishing systems [34,40]. Additionally, lower indexing of African journals in major scientific databases [21] reinforces legitimate anxiety about citation impact and global discoverability. However, integrating DOA awareness into national and institutional research communication strategies is essential to improve adoption and reduce reliance on informal information channels.

### Limitations

The cross-sectional design limits the ability to establish causal relationships between variables, and findings were therefore interpreted as associations rather than causation. The use of convenience sampling may introduce selection bias, as participants were recruited based on availability and willingness to participate. This approach also limits the generalisability of the findings, as the sample may not be fully representative of all researchers in Ghana. Consequently, the results should be interpreted with caution when extending them to the broader research population. Additionally, data were collected via self-reported questionnaires, which may be subject to recall and social desirability biases. Although efforts were made to ensure clarity and anonymity, these biases cannot be entirely ruled out. Despite these limitations, the study employed standardised data collection procedures and appropriate statistical methods, which support the internal validity and reliability of the findings.

## Conclusion

This study revealed that whilst researchers in Ghana possess a general understanding of open access publishing, awareness and knowledge of the Diamond Open Access model remain limited. Higher academic qualifications, particularly at the PhD level, were associated with greater DOA knowledge, whereas early career researchers showed less familiarity. Although participants recognised benefits such as free access and equity in knowledge sharing, concerns about quality, visibility, and sustainability persist, especially among senior academics. The findings highlight the urgent need for policy and institutional reforms to strengthen the adoption of DOA publishing in Ghana. Universities and research institutions should develop comprehensive OA policies that explicitly include DOA models, ensuring sustainable funding and infrastructural support. Academic libraries must be empowered to lead awareness campaigns, provide training, and integrate DOA education into research capacity building programmes. National research agencies and funding bodies should also incentivise DOA publishing through grants and recognition systems that value equity-driven scholarly communication. Such coordinated reforms can foster a more inclusive, transparent, and sustainable research environment across Ghana and the wider African academic landscape.

## Supporting information

S1 Fig Key characteristics of Diamond Open Access journals. (TIFF)

S2 Fig Source of Information of Diamond Open Access journals. (TIFF)

S3 Table Factor analysis of perceived benefits. (DOCX)

S4 Table Factor analysis of perceived challenges. (DOCX)

S5 Fig ANOVA analysis of perceived benefits and challenges of Diamond Open Access publishing based on demographic variables. (TIFF)

S6 Table ANOVA analysis of perceived benefits of Diamond Open Access publishing based on demographic variables. (DOCX)
