# Supplementary material for: Diamond Open Access publishing practices among researchers in Ghana
Source: PLoS One. 2026 Aug 26;21(8):e0356546. doi: 10.1371/journal.pone.0356546 (PMC13513921; doi:10.1371/journal.pone.0356546)
Supplement: Supplementary file 1 [file pone.0356546.s007.pdf]

Comments to the Author

1. Is the manuscript technically sound, and does the data support the conclusions?

Selected response: Yes

Rationale:
The manuscript presents a technically sound cross-sectional survey addressing an important and underexplored topic in scholarly communication. The study design is appropriate for the stated objectives, the sample size (n = 237) is adequate, and the analytical approach aligns with the research questions. The conclusions are generally supported by the descriptive and inferential results, particularly regarding low awareness and knowledge of Diamond Open Access (DOA) and the influence of academic qualification and rank.

However, the use of convenience sampling limits generalizability, and this limitation should be more explicitly emphasized when interpreting national-level implications.

2. Has the statistical analysis been performed appropriately and rigorously?

Selected response: Yes

Rationale:
The statistical analyses are appropriate and competently executed. The authors employ a comprehensive analytical strategy including descriptive statistics, Fisher's exact tests, t-tests, ANOVA, factor analysis, and logistic regression. Model diagnostics (Hosmer–Lemeshow test and VIF) are reported and indicate acceptable model fit and no multicollinearity.

Minor improvements could include:

- Explicit justification for the dichotomization of "knowledge" into poor vs. good categories
- Clarification of how factor score means were interpreted in ANOVA analyses

These issues do not undermine the validity of the findings but would improve transparency.

3. Have the authors made all data underlying the findings fully available?

Selected response: No

Rationale:
There is an inconsistency regarding data availability. While the manuscript states that "all

relevant data are within the manuscript and its Supporting Information files," the "Availability of data and materials" section later indicates that data are available **upon reasonable request**, which does **not comply with PLOS ONE's Data Policy**.

To meet PLOS requirements, the authors should:

- Deposit the anonymized dataset in a public repository (e.g., Zenodo, Figshare, OSF), or
- Clearly explain and justify any ethical or legal restrictions preventing public sharing

This issue **must be resolved prior to acceptance**.

4. Is the manuscript presented in an intelligible fashion and written in standard English?

Selected response: Yes

Rationale:
The manuscript is generally well written, coherent, and understandable. The structure follows standard scientific conventions, and the arguments are logically developed. Minor grammatical issues, typographical inconsistencies (e.g., capitalization of "Diamond Open Access," repetition of section headings, and occasional spacing errors), and some redundancy in the Discussion section should be corrected during revision.

These issues are minor and do not impede comprehension.

5. Review Comments to the Author

General Comments:
This manuscript addresses an important gap in the literature by empirically examining awareness, perceptions, and knowledge of Diamond Open Access publishing among researchers in Ghana. The topic is timely, relevant, and aligned with PLOS ONE's scope and mission of advancing open and equitable science. The study is methodologically sound, clearly reported, and makes a valuable contribution, particularly within the African scholarly communication context.

Major Points for Revision:

1. **Data Availability Compliance (Mandatory):**
   The Data Availability Statement must be corrected to align with PLOS ONE policy. "Available upon reasonable request" is insufficient. The authors should deposit the anonymized dataset in a recognized public repository or clearly justify any restrictions.

2. **Sampling and Generalizability:**
   While convenience sampling is acceptable, the Discussion should more explicitly caution against generalizing findings to all Ghanaian researchers and clarify the potential for selection bias.

3. **Ethics Statement Clarification:**
   The study involves human participants via a survey. Although minimal risk is involved, the ethics section should explicitly state whether institutional ethical approval was obtained or formally waived, consistent with PLOS human-subject research requirements.

4. **Measurement Transparency:**
   Additional detail is recommended on how “knowledge of DOA” was operationalized and dichotomized. Providing a brief description or example items (possibly in supplementary material) would enhance reproducibility.

Minor Suggestions:

- Streamline overlapping discussion points on senior vs. junior researchers
- Standardize terminology (e.g., DOA vs. Diamond OA)
- Improve figure and table captions to be more self-explanatory

Overall, the manuscript is **well executed** and, following minor to moderate revisions, would be suitable for publication.

6. Do you want your identity to be public for this peer review?

Selected response: No

Confidential to Editor

1. Competing interests

Response:
I have no competing interests.

2. Assistance in preparing this review

Response:
No, I did not receive any assistance in preparing this review.

3. If accepted, should this submission be highlighted on the PLOS ONE website?

Selected response: Yes

Reason (optional):
The study addresses an underrepresented geographic context and contributes meaningfully to global discussions on equitable open access publishing, which may be of interest to a broad international readership.

Web of Science Reviewer Recognition

You may opt in or out based on your personal preference.

ORCID Reviewer Credit

Consent may be granted if you wish the review to appear on your ORCID record.
