# Supplementary material for: Diamond Open Access publishing practices among researchers in Ghana
Source: PLoS One. 2026 Aug 26;21(8):e0356546. doi: 10.1371/journal.pone.0356546 (PMC13513921; doi:10.1371/journal.pone.0356546)
Supplement: Supplementary file 2 [file pone.0356546.s003.docx]

**S1 Table**

| **Variable** | **Factor loading** | **Eigen values** | **% of variance** | **Cronbach’s alpha** |
| --- | --- | --- | --- | --- |
| Visibility and Dissemination |  | 5.00179 | 50.02 | 0.8819 |
| Publishing in Diamond Open Access journals enhance reputation of a researcher’s work | 0.711 |  |  |  |
| Diamond Open Access publishing reduces financial barriers for researchers in developing countries | 0.664 |  |  |  |
| Diamond Open Access journals attract high-quality submissions from wider range of researchers | 0.709 |  |  |  |
| Diamond Open Access aligns well with principles of open science and knowledge sharing | 0.715 |  |  |  |
| Diamond Open Access publishing can lead to increased citations for research articles | 0.778 |  |  |  |
| Universities can benefit from supporting and promoting Diamond Open Access journals | 0.719 |  |  |  |
| Diamond Open Access has potential to make scholarly communication more equitable for researcher’s globally | 0.819 |  |  |  |
| Diamond Open Access publishing can encourage collaboration among researchers from different countries | 0.606 |  |  |  |
| Diamond Open Access journals can foster innovation and advancement in research fields | 0.744 |  |  |  |
