# Supplementary material for: Diamond Open Access publishing practices among researchers in Ghana
Source: PLoS One. 2026 Aug 26;21(8):e0356546. doi: 10.1371/journal.pone.0356546 (PMC13513921; doi:10.1371/journal.pone.0356546)
Supplement: Supplementary file 3 [file pone.0356546.s004.docx]

**S2 Table**

| **Variable** | **Factor loading** | **Eigen values** | **% of variance** | **Cronbach’s alpha** |
| --- | --- | --- | --- | --- |
| Quality Concern |  | 4.31155 | 43.12 | 0.8819 |
| Long-term sustainability of Diamond Open Access journals is uncertain | 0.6371 |  |  |  |
| Difficulty in identifying reputable Diamond Open Access journals poses challenger for researchers | 0.6710 |  |  |  |
| Lack of financial incentives through article processing charges (APCs) discourages quality submissions to DOA Journals | 0.6404 |  |  |  |
| Diamond Open Access journals may struggle to build strong readership base due to lower visibility compared to traditional journals | 0.6939 |  |  |  |
| Lack of awareness and understanding of DOA among researchers can hinder wider adoption | 0.7418 |  |  |  |
| Infrastructure and technological requirements for effectively managing Diamond Open Access Journals can be significant hurdle | 0.7271 |  |  |  |
| Encouraging a cultural shift towards open-access publishing within academia can be slow | 0.6790 |  |  |  |
| Predatory journals posing Diamond Open Access can be a concern for researchers | 0.5340 |  |  |  |
| Potential bias against research published in Diamond Open Access journal exist within academic community | 0.6084 |  |  |  |
