# Supplementary material for: Diamond Open Access publishing practices among researchers in Ghana
Source: PLoS One. 2026 Aug 26;21(8):e0356546. doi: 10.1371/journal.pone.0356546 (PMC13513921; doi:10.1371/journal.pone.0356546)
Supplement: Supplementary file 4 [file pone.0356546.s006.docx]

**S3 Table**

| **Visibility and Dissemination** |  |  |  |
| --- | --- | --- | --- |
| **Variables** | **Mean** | **F-value** | **p-Value** |
| **Age (years)** | | | |
| 18–34 | 2.202 | 1.93 | 0.214 |
| 35–49 | 2.363 |  |  |
| 50–60 | 2.511 |  |  |
| Above 60 | 2.850 |  |  |
| **Type of institution** | | | |
| University | 2.850 | 0.99 | 0.400 |
| Colleges | 2.050 |  |  |
| Medical Centres | 2.386 |  |  |
| Others | 2.370 |  |  |
| **Gender** | | | |
| Male | 2.302 | 0.03 | 0.852 |
| Female | 2.284 |  |  |
| **Current rank** | | | |
| Graduate Student | 2.124 | 5.01 | 0.002 |
| Lecturer/Assistant Lecturer | 2.331 |  |  |
| Others | 2.519 |  |  |
| Professor | 2.845 |  |  |
| **Highest academic qualification** | | | |
| Bachelor’s degree | 2.145 | 3.08 | 0.028 |
| Master’s degree | 2.295 |  |  |
| PhD | 2.498 |  |  |
| Post-doctoral | 2.538 |  |  |
