# Supplementary material for: Diamond Open Access publishing practices among researchers in Ghana
Source: PLoS One. 2026 Aug 26;21(8):e0356546. doi: 10.1371/journal.pone.0356546 (PMC13513921; doi:10.1371/journal.pone.0356546)
Supplement: Supplementary file 5 [file pone.0356546.s009.docx]

***Table 1: Point by point response responses to comments***

| **S/N** | **COMMENTS** | **RESPONSE** | **REFERENCE** |
| --- | --- | --- | --- |
| **Second Reviewer’s comment** | | | |
| 1. | Data Availability Compliance (Mandatory):  The Data Availability Statement must be corrected to align with PLOS ONE policy. “Available upon reasonable request” is insufficient. The authors should deposit the anonymised dataset in a recognised public repository or clearly justify any restrictions. | All comments have been addressed | Page 25  line 414 |
| 2. | Sampling and Generalisability:  While convenience sampling is acceptable, the Discussion should more explicitly caution against generalising findings to all Ghanaian researchers and clarify the potential for selection bias | All comments have been addressed | Page 24  line 386-396 |
| 3. | Ethics Statement Clarification:  The study involves human participants via a survey. Although minimal risk is involved, the ethics section should explicitly state whether institutional ethical approval was obtained or formally waived, consistent with PLOS human-subject research requirements | All comments have been addressed | Page 9  line 177-183 |
| 4. | Measurement Transparency:  Additional detail is recommended on how “knowledge of DOA” was operationalised and dichotomised. Providing a brief description or example items (possibly in supplementary material) would enhance reproducibility | All comments have been addressed |  |
| 5. | Streamline overlapping discussion points on senior vs. junior researchers | All comments have been addressed | Page 23  line 366-378 |
| 6. | Standardise terminology (e.g., DOA vs. Diamond OA) | All comments have been addressed | Changes made across the entire manuscript |
| 7. | Improve figure and table captions to be more self-explanatory | All comments have been addressed | Page 14  line 248 |
| **Third Reviewer’s comments** | | | |
| 8. | The different OA types of gold, green and diamond should be further elaborated in introduction. A clearer explanation on these models are essential to understand the research gap. Are there any other studies reporting Ghanaian's perception of OA other than Atiso et al? | All comments have been addressed | Page 4  Line 68-73 |
| 9. | Although the questionnaire was created with references to the literature and the expert opinion and underwent pilot testing to guarantee the clarity, the manuscript lacks enough proof of formal validation. Pilot testing among 10 faculty members primarily assesses clarity and structure but does not establish content validity, construct validity, or reliability. In the case of a quantitative survey study, particularly where the instrument is a measure of perceptions, attitudes or knowledge construct, the evidence of psychometric properties, especially assessment of content validity eg CVI , reliability testing eg Cronbachs alpha, and factor analysis is needed.  The authors should clarify:  The instrument was based on an existing validated tool or not.  In case it was developed recently, what were the formal validation procedures that were carried out.  Statistical reliability of the final instrument.  This aspect needs to be revised greatly before the manuscript can be taken into consideration by publication. | All comments have been addressed | Page 9 line 172-173  Page 19 line 278-282 |
| 10. | In general, statistical analysis is comprehensive but factor analysis procedure needs more detailed explanation. | All comments have been addressed | Page 11  line 196-214 |
| 11. | The manuscript didn't indicate that formal ethical approval was received from any institutional review board or ethics committee. Even in the case of the minimal-risk studies involving questionnaires like this study, researchers usually have to get ethical approval or a formal exemption letter of the concerned institutional body.  Although this study might seem to be not very risky, ethical oversight would make sure that the ethical and international research standards were followed, such as informed consent, confidentiality, and protection of the data. The authors ought to state whether they received ethical approval or exemption and the number they received in the case of the former. | All comments have been addressed | Page 9  line 177-183 |
