# Supplementary material for: Diamond Open Access publishing practices among researchers in Ghana
Source: PLoS One. 2026 Aug 26;21(8):e0356546. doi: 10.1371/journal.pone.0356546 (PMC13513921; doi:10.1371/journal.pone.0356546)
Supplement: Peer review history

# Peer review: Decision Letter 0

**DOI:** 10.1371/journal.pone.0356546.r001

**Authors:** Tatchalerm Sudhipongpracha

8 Apr 2026

Dear Dr. Agyei,

Thank you for submitting your manuscript to PLOS ONE. After careful consideration, we feel that it has merit but does not fully meet PLOS ONE’s publication criteria as it currently stands. Therefore, we invite you to submit a revised version of the manuscript that addresses the points raised during the review process.

Please submit your revised manuscript by May 23 2026 11:59PM. If you will need more time than this to complete your revisions, please reply to this message or contact the journal office at plosone@plos.org. When you're ready to submit your revision, log on to https://www.editorialmanager.com/pone/ and select the 'Submissions Needing Revision' folder to locate your manuscript file.

- A letter that responds to each point raised by the academic editor and reviewer(s). You should upload this letter as a separate file labeled 'Response to Reviewers'.
- A marked-up copy of your manuscript that highlights changes made to the original version. You should upload this as a separate file labeled 'Revised Manuscript with Track Changes'.
- An unmarked version of your revised paper without tracked changes. You should upload this as a separate file labeled 'Manuscript'.

If you would like to make changes to your financial disclosure, please include your updated statement in your cover letter. Guidelines for resubmitting your figure files are available below the reviewer comments at the end of this letter.

If applicable, we recommend that you deposit your laboratory protocols in protocols.io to enhance the reproducibility of your results. Protocols.io assigns your protocol its own identifier (DOI) so that it can be cited independently in the future. For instructions see: https://journals.plos.org/plosone/s/submission-guidelines#loc-laboratory-protocols. Additionally, PLOS ONE offers an option for publishing peer-reviewed Lab Protocol articles, which describe protocols hosted on protocols.io. Read more information on sharing protocols at https://plos.org/protocols?utm_medium=editorial-email&utm_source=authorletters&utm_campaign=protocols.

As the corresponding author, your ORCID iD is verified in the submission system and will appear in the published article. PLOS supports the use of ORCID, and we encourage all coauthors to register for an ORCID iD and use it as well. Please encourage your coauthors to verify their ORCID iD within the submission system before final acceptance, as unverified ORCID iDs will not appear in the published article. *Only*  the individual author can complete the verification step; PLOS staff *cannot*  verify ORCID iDs on behalf of authors.

We look forward to receiving your revised manuscript.

Kind regards,

Tatchalerm Sudhipongpracha

Academic Editor

PLOS One

Journal Requirements:

When submitting your revision, we need you to address these additional requirements.

1. Please ensure that your manuscript meets PLOS ONE's style requirements, including those for file naming. The PLOS ONE style templates can be found at

https://journals.plos.org/plosone/s/file?id=wjVg/PLOSOne_formatting_sample_main_body.pdf and https://journals.plos.org/plosone/s/file?id=ba62/PLOSOne_formatting_sample_title_authors_affiliations.pdf

2. Please include captions for your Supporting Information files at the end of your manuscript, and update any in-text citations to match accordingly. Please see our Supporting Information guidelines for more information: http://journals.plos.org/plosone/s/supporting-information.

3. If the reviewer comments include a recommendation to cite specific previously published works, please review and evaluate these publications to determine whether they are relevant and should be cited. There is no requirement to cite these works unless the editor has indicated otherwise.

[Note: HTML markup is below. Please do not edit.]

Reviewers' comments:

Reviewer's Responses to Questions

**Comments to the Author**

1. Is the manuscript technically sound, and do the data support the conclusions?

Reviewer #1: Yes

Reviewer #2: Yes

Reviewer #3: No

**********

2. Has the statistical analysis been performed appropriately and rigorously?

Reviewer #1: Yes

Reviewer #2: Yes

Reviewer #3: Yes

**********

3. Have the authors made all data underlying the findings in their manuscript fully available?

The PLOS Data policy

Reviewer #1: Yes

Reviewer #2: No

Reviewer #3: Yes

**********

4. Is the manuscript presented in an intelligible fashion and written in standard English?

Reviewer #1: Yes

Reviewer #2: Yes

Reviewer #3: Yes

**********

Reviewer #1: The methodological and statistical methods of the paper are sound. The writing is satisfactory and the references and figures and tables are good. Therefore, the paper is in my opinion acceptable for publication.

Reviewer #2: General Comments:

This manuscript addresses an important gap in the literature by empirically examining awareness, perceptions, and knowledge of Diamond Open Access publishing among researchers in Ghana. The topic is timely, relevant, and aligned with PLOS ONE’s scope and mission of advancing open and equitable science. The study is methodologically sound, clearly reported, and makes a valuable contribution, particularly within the African scholarly communication context.

Major Points for Revision:

1. Data Availability Compliance (Mandatory):

The Data Availability Statement must be corrected to align with PLOS ONE policy. “Available upon reasonable request” is insufficient. The authors should deposit the anonymized dataset in a recognized public repository or clearly justify any restrictions.

2. Sampling and Generalizability:

While convenience sampling is acceptable, the Discussion should more explicitly caution against generalizing findings to all Ghanaian researchers and clarify the potential for selection bias.

3. Ethics Statement Clarification:

The study involves human participants via a survey. Although minimal risk is involved, the ethics section should explicitly state whether institutional ethical approval was obtained or formally waived, consistent with PLOS human-subject research requirements.

4. Measurement Transparency:

Additional detail is recommended on how “knowledge of DOA” was operationalized and dichotomized. Providing a brief description or example items (possibly in supplementary material) would enhance reproducibility.

Minor Suggestions:

• Streamline overlapping discussion points on senior vs. junior researchers

• Standardize terminology (e.g., DOA vs. Diamond OA)

• Improve figure and table captions to be more self-explanatory

Overall, the manuscript is well executed and, following minor to moderate revisions, would be suitable for publication.

Reviewer #3: 1. The different OA types of gold, green and diamond should be further elaborated in introduction. A clearer explanation on these models are essential to understand the research gap. Are there any other studies reporting Ghanaian's perception of OA other than Atiso et al?

2. Although the questionnaire was created with references to the literature and the expert opinion and underwent pilot testing to guarantee the clarity, the manuscript lacks enough proof of formal validation. Pilot testing among 10 faculty members primarily assesses clarity and structure but does not establish content validity, construct validity, or reliability.

In the case of a quantitative survey study, particularly where the instrument is a measure of perceptions, attitudes or knowledge construct, the evidence of psychometric properties, especially assessment of content validity eg CVI , reliability testing eg Cronbachs alpha, and factor analysis is needed.

The authors should clarify:

The instrument was based on an existing validated tool or not.

In case it was developed recently, what were the formal validation procedures that were carried out.

Statistical reliability of the final instrument.

This aspect needs to be revised greatly before the manuscript can be taken into consideration by publication.

3. In general, statistical analysis is comprehensive but factor analysis procedure needs more detailed explanation.

4. The manuscript didn't indicate that formal ethical approval was received from any institutional review board or ethics committee. Even in the case of the minimal-risk studies involving questionnaires like this study, researchers usually have to get ethical approval or a formal exemption letter of the concerned institutional body.

Although this study might seem to be not very risky, ethical oversight would make sure that the ethical and international research standards were followed, such as informed consent, confidentiality, and protection of the data. The authors ought to state whether they received ethical approval or exemption and the number they received in the case of the former.

**********

what does this mean?). If published, this will include your full peer review and any attached files.

If you choose “no”, your identity will remain anonymous but your review may still be made public.

**Do you want your identity to be public for this peer review?**  For information about this choice, including consent withdrawal, please see our Privacy Policy

Reviewer #1: No

Reviewer #2: No

Reviewer #3: No

**********

[NOTE: If reviewer comments were submitted as an attachment file, they will be attached to this email and accessible via the submission site. Please log into your account, locate the manuscript record, and check for the action link "View Attachments". If this link does not appear, there are no attachment files.]

To ensure your figures meet our technical requirements, please review our figure guidelines: https://journals.plos.org/plosone/s/figures

You may also use PLOS’s free figure tool, NAAS, to help you prepare publication quality figures: https://journals.plos.org/plosone/s/figures#loc-tools-for-figure-preparation.

NAAS will assess whether your figures meet our technical requirements by comparing each figure against our figure specifications.

Attachment Submitted filename: Peer review report 22-1-2026.pdf

# Author comment: Author response to Decision Letter 1

**DOI:** 10.1371/journal.pone.0356546.r002

23 Apr 2026

All comments have been responded to and uploaded as "Response to Reviewers".

Attachment Submitted filename: Response to Reviewers.docx

# Peer review: Decision Letter 1

**DOI:** 10.1371/journal.pone.0356546.r003

**Authors:** Tatchalerm Sudhipongpracha

5 Aug 2026

Diamond Open Access Publishing Practices among Researchers in Ghana

PONE-D-25-68379R1

Dear Dr. Agyei,

We’re pleased to inform you that your manuscript has been judged scientifically suitable for publication and will be formally accepted for publication once it meets all outstanding technical requirements.

Within one week, you’ll receive an e-mail detailing the required amendments. When these have been addressed, you’ll receive a formal acceptance letter and your manuscript will be scheduled for publication.

An invoice will be generated when your article is formally accepted. Please note, if your institution has a publishing partnership with PLOS and your article meets the relevant criteria, all or part of your publication costs will be covered. Please make sure your user information is up-to-date by logging into Editorial Manager at Editorial Manager® and clicking the ‘Update My Information' link at the top of the page. For questions related to billing, please contact billing support.

If your institution or institutions have a press office, please notify them about your upcoming paper to help maximize its impact. If they’ll be preparing press materials, please inform our press team as soon as possible -- no later than 48 hours after receiving the formal acceptance. Your manuscript will remain under strict press embargo until 2 pm Eastern Time on the date of publication. For more information, please contact onepress@plos.org.

Kind regards,

Tatchalerm Sudhipongpracha

Academic Editor

PLOS One

Additional Editor Comments (optional):

Reviewers' comments:

Reviewer's Responses to Questions

**Comments to the Author**

Reviewer #1: All comments have been addressed

Reviewer #2: All comments have been addressed

**********

2. Is the manuscript technically sound, and do the data support the conclusions?<br/><br/>The manuscript must describe a technically sound piece of scientific research with data that supports the conclusions. Experiments must have been conducted rigorously, with appropriate controls, replication, and sample sizes. The conclusions must be drawn appropriately based on the data presented.

Reviewer #1: Yes

Reviewer #2: Yes

**********

3. Has the statistical analysis been performed appropriately and rigorously?

Reviewer #1: Yes

Reviewer #2: Yes

**********

4. Have the authors made all data underlying the findings in their manuscript fully available?<br/><br/>The ?>PLOS Data policy

Reviewer #1: Yes

Reviewer #2: Yes

**********

5. Is the manuscript presented in an intelligible fashion and written in standard English?<br/><br/>PLOS ONE does not copyedit accepted manuscripts, so the language in submitted articles must be clear, correct, and unambiguous. Any typographical or grammatical errors should be corrected at revision, so please note any specific errors here.

Reviewer #1: Yes

Reviewer #2: Yes

**********

Reviewer #1: it is now acceptable for publication, because of the word limit I have to continue writing even though I have nothing else to say

Reviewer #2: Thank you for carefully addressing the reviewer comments. The revised manuscript has improved substantially and addresses the major concerns raised in the previous review round.

The authors have clarified the ethical approval process and informed consent procedures, strengthened the discussion of study limitations related to convenience sampling and selection bias, and provided additional methodological detail regarding the measurement and categorization of Diamond Open Access knowledge. These revisions improve the transparency, reproducibility, and overall quality of the manuscript.

The statistical analyses are appropriate and clearly reported. The discussion and conclusions are balanced and generally supported by the findings. The manuscript addresses an important and underexplored topic in scholarly communication and contributes valuable evidence from the African research context.

The revised Data Availability Statement now indicates that all relevant data are included in the paper and Supporting Information files. Provided that the underlying anonymized dataset is available as stated and complies with PLOS ONE data-sharing requirements, I have no further substantive concerns.

Overall, I believe the manuscript is suitable for publication in PLOS ONE.

**********

what does this mean?). If published, this will include your full peer review and any attached files.

If you choose “no”, your identity will remain anonymous but your review may still be made public.

**Do you want your identity to be public for this peer review?**  For information about this choice, including consent withdrawal, please see our Privacy Policy

Reviewer #1: No

Reviewer #2: No

**********

# Editor report: Acceptance letter

**DOI:** 10.1371/journal.pone.0356546.r004

**Authors:** Tatchalerm Sudhipongpracha

PONE-D-25-68379R1

PLOS One

Dear Dr. Agyei,

I'm pleased to inform you that your manuscript has been deemed suitable for publication in PLOS One. Congratulations! Your manuscript is now being handed over to our production team.

At this stage, our production department will prepare your paper for publication. This includes ensuring the following:

* All references, tables, and figures are properly cited

* All relevant supporting information is included in the manuscript submission,

* There are no issues that prevent the paper from being properly typeset

You will receive further instructions from the production team, including instructions on how to review your proof when it is ready. Please keep in mind that we are working through a large volume of accepted articles, so please give us a few days to review your paper and let you know the next and final steps.

Lastly, if your institution or institutions have a press office, please let them know about your upcoming paper now to help maximize its impact. If they'll be preparing press materials, please inform our press team within the next 48 hours. Your manuscript will remain under strict press embargo until 2 pm Eastern Time on the date of publication. For more information, please contact onepress@plos.org.

You will receive an invoice from PLOS for your publication fee after your manuscript has reached the completed accept phase. If you receive an email requesting payment before acceptance or for any other service, this may be a phishing scheme. Learn how to identify phishing emails and protect your accounts at https://explore.plos.org/phishing.

If we can help with anything else, please email us at customercare@plos.org.

Thank you for submitting your work to PLOS One and supporting open access.

Kind regards,

PLOS ONE Editorial Office Staff

on behalf of

Dr. Tatchalerm Sudhipongpracha

Academic Editor

PLOS One
